# Electroacupuncture Activates Neuroplasticity in the Motor Cortex and Corticospinal Tract via the mTOR Pathway in a Rat P-MCAO Model

**DOI:** 10.1155/2022/3470685

**Published:** 2022-11-14

**Authors:** You Zhang, Ya-Long Yin, Zi-Yan Jin, Qi-Ping Hu, Xin-gui Wu

**Affiliations:** ^1^Department of Traditional Chinese Medicine, First Affiliated Hospital of Guangxi Medical University, Nanning, Guangxi Zhuang Autonomous Region, 530021, China; ^2^Department of Cell Biology and Genetics, School of Pre-Clinical Medicine, Guangxi Medical University, Nanning, Guangxi Zhuang Autonomous Region, 530021, China

## Abstract

Electroacupuncture (EA) combines traditional Chinese medicine acupuncture theory with modern scientific technology. It is a promising therapy for the treatment of cerebrovascular diseases such as cerebral infarction. A large number of clinical studies have shown that EA promotes recovery of neurological function after cerebral infarction, however, the underlying mechanisms behind its effects remain unclear. We tested whether EA stimulation of the Zusanli (ST36) and Neiguan (PC6) acupoints activates neuroplasticity in rats with ischemic stroke and whether this involves the regulation of axonal regeneration through the mTOR pathway. 24 h after permanent middle cerebral artery occlusion (p-MCAO) in rats, EA treatment was started for 20 min, daily, for 14 days. We found that EA significantly reduced Modified Neurological Severity Scores (mNSS), cerebral infarct volume, and apoptosis of neuronal cells. EA also significantly increased the expression of the neuroplasticity-associated proteins GAP-43 and SYN and upregulated the phosphorylation levels of AKT, mTOR, S6, and PTEN to promote CST axon sprouting in the spinal cord at C1–C4 levels. The positive effects of EA were blocked by the administration of the mTOR inhibitor Rapamycin. In short, we found that EA of the Zusanli (ST36) and Neiguan (PC6) acupoints in p-MCAO rats induced neuroprotective and neuroplastic effects by regulating the mTOR signaling pathway. It promoted neuroplasticity activated by axon regeneration in the contralateral cortex and corticospinal tract. Activation of such endogenous remodeling is conducive to neurological recovery and may help explain the positive clinical effects seen in patients with infarcts.

## 1. Introduction

Cerebral infarction, commonly known as “stroke”, is a cerebrovascular disease caused by cerebral ischemia and hypoxia that results in cell necrosis and subsequent neurological dysfunction [1]. The condition is associated with a poor overall prognosis, high morbidity, disability, recurrence, and mortality; as such, it is one of the leading causes of neurological dysfunction and death in the elderly and is of great research and clinical interest [2]. The basic elements of neural repair including axonal sprouting, dendritic branching, synaptogenesis, neurogenesis, and glial scar formation can be observed in animal models of cerebral infarction; the recovery of neural function after cerebral infarction requires synaptogenesis and axonal sprouting to activate neuroplasticity in areas of cerebral ischemic injury [3]. The corticospinal tract (CST) is a key brain and spinal cord motor pathway and an important target for neural repair [4]. The limited ability of adult mammalian neurons to regenerate damaged axons is due to the developmental loss of the inherent ability to initiate axonal growth. Although unable to regenerate lost axons, undamaged CST neurons carry some capacity for local axonal growth or sprouting; unfortunately, this endogenous regenerative capacity is limited and without additional intervention, it is difficult to rely on compensatory recovery after injury to return the organism to normal function [5]. Our goal was to exploit therapeutic techniques to stimulate and amplify endogenous recovery mechanisms to improve neurological dysfunction.

Electroacupuncture (EA) is a form of acupuncture that combines traditional acupuncture and electrical stimulation and is used as a complementary therapy in poststroke patients in China. The positive effects of electroacupuncture treatment following stroke injury have been demonstrated in several studies and included the promotion of neurological recovery and improved patient quality of life [6–8]. Effects such as the promotion of vascular [9] and nerve regeneration [10], and the release of neuroprotective neurotrophic factors [11] have been reported. However, the underlying regulatory mechanisms of electroacupuncture in the treatment of stroke remain unclear.

Work with animal models has shown that axonal sprouting in the brain establishes new connection patterns during recovery and that axonal sprouting occurs in the contralateral cortex sensorimotor projections to the cervical medulla. This axonal sprouting enters the part of the cervical medulla that has lost corticospinal projections from the stroke-affected region and promotes recovery of motor function [12]. In several CNS injury models, upregulation of mTOR was sufficient to cause significant axonal growth [13].

mTOR is a 289 kDa serine/threonine protein kinase that is active throughout development and is critical for normal brain and neural circuit development [14]. In previous studies, EA was found to be neuroprotective via activation of the mTOR pathway [15, 16]. Activation of the mTOR pathway promotes CST axonal growth and presynaptic synapse formation; blockade of the mTOR pathway inhibits CST axonal growth [17]. mTOR activation may be triggered by stimulus-related calcium inward flow, as evidenced by elevated ipsilateral pS6 protein levels following electrical stimulation of the motor cortex and an increase in the number of pS6-positive neurons in layer 5 of the cerebral motor cortex [18]. Therefore, we hypothesized that EA may promote axonal regeneration after ischemic stroke and exert neuroprotective effects by activating the mTOR pathway.

In this study, we investigated whether electroacupuncture stimulation of the Zusanli (ST36) and Neiguan (PC6) acupoints regulates axonal regeneration in the contralateral cortex and CST after ischemic brain injury through the mTOR signaling pathway, and activates neuroplasticity to achieve neuroprotective effects. This study provides new insights into the molecular mechanisms of electroacupuncture in the treatment of ischemic stroke.

## 2. Materials and Methods

### 2.1. Permanent Middle Cerebral Artery Occlusion Model

The Permanent Middle Cerebral Artery Occlusion (p-MCAO) rat model was induced via the modified Longa thread occlusion method [19]. First, each rat was fasted overnight (12–14 h), then anesthetized by an intraperitoneal injection of Ketamine (60 mg/kg) + Xylazine (8 mg/kg). The animal was then immobilized in supine, disinfected, and a midline incision was made in the neck to expose the right common carotid (CCA), internal carotid (ICA), and external carotid (ECA) arteries. A surgical nylon suture (diameter 0.37 mm; Guangzhou Jialing Biotechnology Co., Ltd, China) was inserted into the bifurcation, 5 mm from the ICA. The right middle cerebral artery (MCA) blood flow was occluded by advancing in parallel along the ICA. The ICA and CCA were ligated to fix the thread bolt and the incision was sutured layer by layer after hemostasis and disinfection. Body temperature was monitored rectally and maintained at 37°C with an electric blanket during and after the surgery. The nylon suture remained in place in rats undergoing p-MCAO until 14 days after occlusion. Animals in the sham group underwent the same procedure but the ligation of blood vessels was not performed.

### 2.2. Animals and Groups

Specific-Pathogen Free (SPF)-grade healthy male Sprague Dawley (SD) rats with an average weight of (280 ± 50 g) were selected from a colony at the Experimental Animal Center of Guangxi Medical University. The animals were raised in the Animal Experimental Center of Guangxi Medical University, with ad libitum water intake and feeding. The ambient room temperature was 24 ± 4°C and the average relative humidity was 55%; light and dark were alternated every 12 hours. Fasting animals were allowed to maintain water intake. The experimental scheme was approved by the Animal Ethics Committee of Guangxi Medical University (202103019).

**Figure 1:**
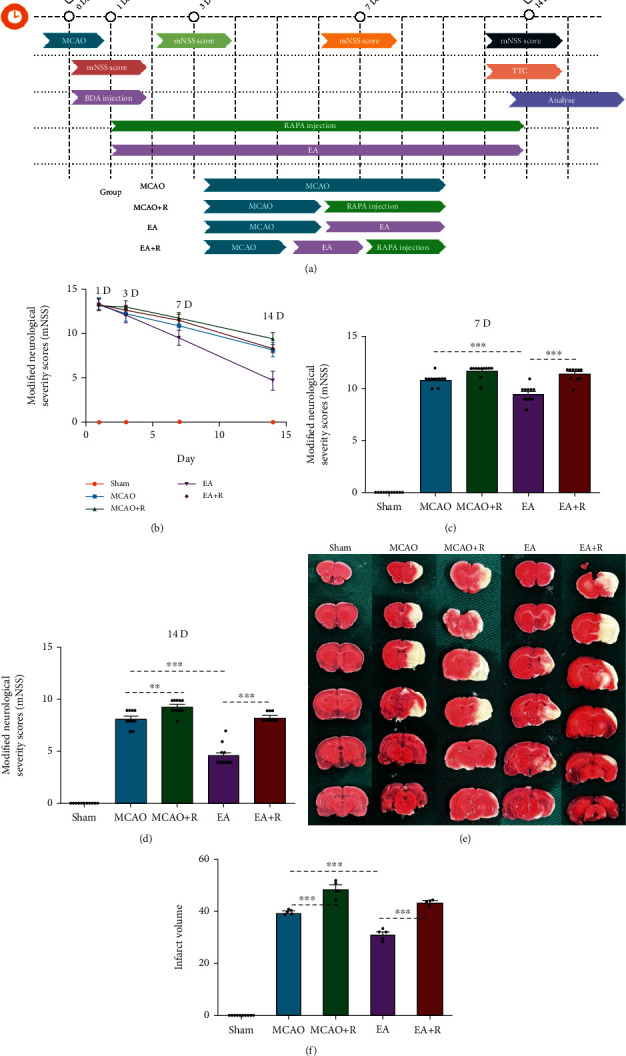
Pathological and behavioral changes 14 days after ischemic injury. (a) Experimental grouping and intervention protocol. (b) Comparison of mNSS scores at different times after ischemic injury (*n* = 10). (c) mNSS score 7 days after ischemic injury. (d) mNSS score 14 days after ischemic injury. (e) 2,3,5-triphenyltetrazolium (TTC) staining showing cerebral infarct volumes in the Sham, p-MCAO, and p-MCAO + R, EA, and EA + R groups (*n* = 10). (f) Bar graph showing the percentage of cerebral infarct volume in the five groups. (^∗^*p* < 0.05, ^∗∗^*p* < 0.01, ^∗∗∗^*p* < 0.001).

As shown in Figure 1(a), 100 rats were randomly divided into 5 groups (*n* = 20 per group) as follows: (i) sham rats underwent a cervical incision and vascular exposure without embolization; (ii) p-MCAO rats received a permanent occlusion in the right MCA; (iii) MCAO + R rats received the same treatment as p-MCAO rats plus rapamycin (3 mg/kg in DMSO, i.p, per 24 hours, Solarbio, China), a specific inhibitor of the mTOR pathway, which was injected intraperitoneally every 24 hours postoperatively for 14 days with the last injection occuring 30 minutes before animal sacrifice; (iv) EA rats received the same treatment as p-MCAO rats plus EA for 20 min following the surgery; (v) EA + R received the same treatment as EA rats with the addition of daily i.p. rapamycin for 14 days as in the p-MCAO + R group.

### 2.3. Anterograde Labeling of CST Axons

24 hours after p-MCAO, 5 rats in each group were selected for anterograde nerve tracing. The rats were anesthetized and placed in a stereotaxic frame (Reward, China). Rectal temperature was kept at 37°C throughout the procedure. The scalp contralateral to the infarction was cut along the midline to expose the contralateral skull. After drilling, BDA (10000 molecular weight, 10% w/v solution in 0.1MPBS; Invitrogen) anterograde tracer was administered using a microsyringe at 7 standard points in the contralateral cortex at a depth of 1.8 mm according to the modified method of Zareen et al. [17]. (AP: anteroposterior, distance from the anterior fontanelle, ML: mediolateral, distance from the midline of the skull, unit mm), stereotactic coordinates were: (0.5, 2.5), (0.5, 3.5), (1.0, 3.5), (1.5, 2.5), (1.5, 3.5), (2.5, 2.5), and (2.5, 3.5). Each location received a total volume of 0.3 *μ*L. Each dose was administered in 0.1 *μ*L every 5 minutes for a total of three doses. We carefully avoided and monitored for intraoperative vascular injury and postoperative infections.

### 2.4. The EA Intervention

We stimulated the Zusanli (ST36) and Neiguan (PC6) points of the bilateral hind and forelimbs, respectively, using an EA apparatus (Model SDZ-II; Hwato, China). Animals were anesthetized with isoflurane gas and immobilized in preparation for EA treatment. Acupuncture needles with a diameter of 0.3 mm were inserted into the bilateral ST36 and PC6 acupoints to a depth of 2 mm and 1 mm, respectively. Stimulation parameters were a dense wave of 2 Hz and intensity of 1 mA. Rats in the sham control, p-MCAO, and pMACO + R groups were only anesthetized and immobilized without EA treatment.

### 2.5. Modified Neurological Severity Scores

The modified neurological severity scores (mNSS) [20] was used to evaluate the degree of neurological impairment at 1, 3, 7, and 14 days after the cerebral infarction. The scores include motor, sensory (visual, tactile, and proprioception), balance, and reflex domains. The degree of neurological impairment is scored 0–18 (0 being least and 18 being the most impaired). The evaluation criteria were categorized as follows: mild injury (1–6), moderate injury (7–12), and severe injury (13–18). Animals scored as moderately injured 1 day after p-MCAO were included in the study.

### 2.6. TTC Staining and Infarct Volume Measurement

The volume of cerebral infarction was measured by 2,3,5-Triphenyltetrazolium Chloride (TTC) staining. Five rats in each group were sacrificed by i.p. anesthesia overdose on the 14th day and utilized for TTC staining. We harvested the whole brain and froze it at -20°C for 15 min. The brain was cut into 6 x 2 mm coronal sections and stained in 2% TTC (Solarbio, China) for 30 min at 37°C, then fixed overnight in 4% paraformaldehyde buffer before imaging. TTC stained sections show normal areas in dark red and the infarcted areas in white. We quantified the infarct volume using ImageJ software.

### 2.7. Nissl Staining

Nissl staining was used to evaluate neuronal damage in the cortex. Rats were sacrificed via excessive i.p. anesthesia. We performed cardiac perfusion with 0.01 M PBS, followed by 250 mL of 4% paraformaldehyde (4°C) as a tissue fixative, and finally removed the contralateral motor cortex. Routine paraffin embedding was performed and 5 *μ*m sections were subsequently generated. Paraffinized sections were soaked in xylene ×3 for 10 min, then dehydrated via an ethanol gradient (95%, 90%, 80%, 70%, and 50% for 5 min each), immersed in Nissl staining solution (Beyotime, China) for 30 min, rinsed quickly with water, ethanol gradient dehydrated, and soaked in xylene ×3 for 2 min. Finally, sections were coverslipped and three randomly selected visual fields were photographed under a microscope. Nissl positive cells were quantified using ImageJ software.

### 2.8. Western Blot Analysis

Protein and synaptic growth-related proteins in the PTEN/AKT/mTOR pathway were detected using Western blotting. Five rats in each group were sacrificed and the total protein was extracted from the contralateral cerebral motor cortex. Brain tissue was dissociated and homogenized in a protein lysate then centrifuged at 12000 g for 30 min at 4°C. The supernatant was collected and stored at -80°C. The protein concentration of each group was quantified using a BCA protein detection kit (Beyotime, China). 70 *μ*g of protein per sample was loaded into a 10% SDS-PAGE gel for electrophoresis, then transferred to a PVDF membrane (Millipore), incubated in a 5% calf serum albumin solution (BSA, Elabscience) at 37°C for 1 hour. Primary antibody solution was added and the membranes were placed on a shaker at 4°C overnight. The antibodies included PTEN (1 : 300, 7974, Santa Cruz Biotech), Phospho-PTEN (1 : 300, 377573, Santa Cruz Biotech), AKT (1 : 1000, 4691 T, Cell Signaling Technology), Phospho-AKT (1 : 1000, 4060 T, Cell Signaling Technology), mTOR (1 : 1000, 2983 T, Cell Signaling Technology), Phospho-mTOR (1 : 1000, 5536S, Cell Signaling Technology), S6 (1 : 1000, 2217S, Cell Signaling Technology), Phospho-S6 (1 : 1000, 4858S, Cell Signaling Technology), GAP-43 (1 : 500, AB5220, Sigma), SYN (1 : 400, S5768, Sigma), *β*-actin (1 : 1000, 4970S, Cell Signaling Technology), and GADPH (1 : 1000, 5174 T, Cell Signaling Technology). On the second day, the membranes were rinsed ×3 with TBST. The corresponding secondary antibody was added and incubated at room temperature for 1 hour: goat-anti-rabbit (1 : 2000, A-1003, Elabscience) or goat-anti-mouse(1 : 5000, A-1001, Elabscience). Protein bands were detected via chemiluminescence substrate (ECL) and the band intensities were quantified by using ImageJ software.

### 2.9. Immunofluorescence

Rats were sacrificed using excessive anesthesia (*n* = 5 per group) and the contralateral motor cortex was harvested following a 4% paraformaldehyde cardiac perfusion. The brain was embedded in OCT and sectioned at 10 *μ*m. Antigen retrieval was performed by placing the sections in preheated citrate buffer for 5 min and then blocked in 5% bovine serum albumin (BSA) in 37°C for 60 min. Sections were then incubated overnight at 4°C with rabbit anti-p-S6 (1 : 200, Cell Signaling Technology) and mouse anti-SYN (1 : 200, Sigma). The following day, the sections were washed in PBS ×3 for 5 min, then incubated in the dark at room temperature × 60 min with secondary antibodies: goat-anti-rabbit Alexa Fluor 488 (1 : 200, 11008, Invitrogen) and goat-anti-mouse Alexa Fluor 594 (1 : 500, A21203, Invitrogen). After PBS washing ×3, the nucleus was stained with 4,6-diamino-2-phenylindole (DAPI) × 15 min. P-S6/SYN double-labeled immunofluorescence staining was the same as above. The fluorescence signal was detected at 200× magnification under a fluorescence microscope (OLYMPUS); we selected three nonoverlapping visual fields per section for analysis. The results show optical density (OD). We found differences in the expression of p-S6 and SYN in the contralateral motor cortex of rats in different groups.

### 2.10. Immunofluorescence of Spinal Cord CST

For biotinylated dextran amine (BDA) immunohistochemical analysis of axon labeling, we embedded the C1–C4 spinal cord regions in OCT as described above and prepared 30 *μ*m sections. These were immersed in 0.3% TritonX-100 for 30 min, then incubated with streptavidin coupled to Alexa Fluor 488 (1 : 500, S11223, Invitrogen) at room temperature for 1 hour. For BDA/p-S6; BDA/SYN double-labeled immunofluorescence staining, we sectioned in the same manner. The primary antibody was rabbit anti-p-S6 (1 : 200, Cell Signaling Technology) and mouse anti-SYN (1 : 200, Cell Signaling Technology). The secondary antibody was Alexa Fluor 488 streptavidin (1 : 500), Alexa Flour 594 donkey-anti-rabbit IgG (1 : 500, A21207, Invitrogen), Alexa Fluor 488 streptavidin, and Alexa Fluor 594 goat-anti-mouse (1 : 500, Invitrogen). Fluorescence was detected using a fluorescent microscope and a two-photon laser scanning microscope (LaVision BioTec, TriM Scope II, Germany). We selected and analyzed three nonoverlapping visual fields from each section. The results showed optical density (OD).

### 2.11. Statistical Analysis

All data were processed using Prism 5 (GraphPad Software). Quantitative data were expressed as mean ± standard deviation. Differences between different groups were tested using a one-way analysis of variance (ANOVA) with Bonferroni correction. Nonnormally distributed data were tested using Dunnett's Test. We determined statistical significance based on a threshold of *α* = 0.05.

## 3. Results

### 3.1. EA Attenuated P-MCAO Neurological Deficit Scores and Brain Infarct Volume

To test if EA exerts a neuroprotective effect, we performed neurological deficit scores at 1, 3, 7, and 14 days after p-MCAO. Differences in neurological deficit scores between the p-MCAO and EA groups at 1 and 3 days after surgery were not statistically significant but after 7 days of intervention, the neurological deficit scores in the EA group were significantly lower compared with the p-MCAO group (*p* < 0.001) (Figures 1(b) and 1(c)). By 14 days of intervention, the reduction of neurological deficit scores was more significant in the EA group compared with the p-MCAO group (*p* < 0.001). In addition, the neurological deficit scores in the E + R group were significantly higher than those in the EA group (*p* < 0.001) (Figure 1(d)). Cerebral infarct volume assessment was performed 14 days after p-MCAO. As shown in Figure 1(e), after staining TTC staining, we found no significant ischemic changes in the Sham group but the other groups had ischemic changes of varying severity. As shown in Figure 1(f), significant ischemic infarct foci appeared after p-MCAO; the infarct volume in the EA group was significantly smaller compared with the p-MCAO group (*p* < 0.001), while the infarct volume in the E + R group was significantly larger than in the EA group (*p* < 0.001).

### 3.2. EA Reduces P-MCAO Neuronal Damage

**Figure 2:**
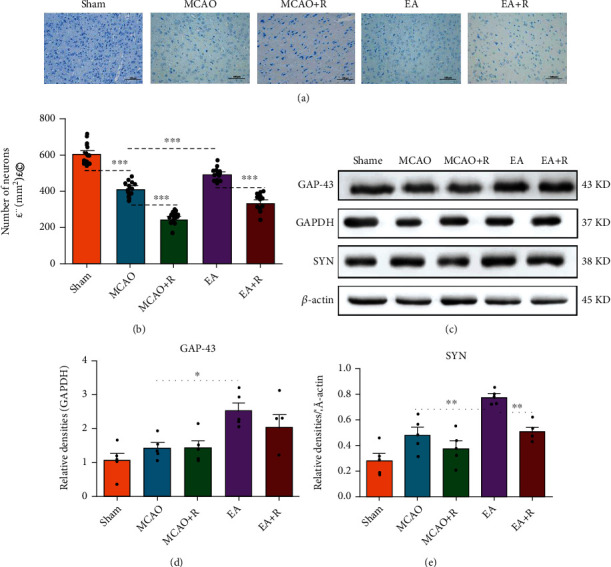
EA activates neuroplasticity-associated proteins to attenuate neuronal damage. (a) Results of different groups of Nissl staining (*n* = 5, Scale bar = 100 *μ*m). (b) Western blotting analysis of the expression levels of GAP-43 and SYN (*n* = 5). (c) Changes in the number of intact neurons in each group. (d) Bar graph showing differences in SYN in the rat robust cerebral cortex. (e) Statistical analysis of GAP-43 expression in the rat robust cerebral cortex (^∗^*p* < 0.05, ^∗∗^*p* < 0.01, ^∗∗∗^*p* < 0.001).

We assessed cortical neuronal damage by quantifying the number of neurons in the cerebral cortex contralateral to the infarct by using Nissl staining (Figure 2(a)). As shown in Figure 2(c), the p-MCAO group had lighter neuronal staining, looser cellular arrangement, and fewer neurons compared with the Sham group (*p* < 0.001). In contrast, after 14 days of EA intervention, the number of degenerated neurons was significantly reduced and the number of intact neurons was increased in the EA group compared with the p-MCAO group (*p* < 0.001). Rapamycin inhibited the effect of EA and reduced the number of neurons compared to the EA-only group (*p* < 0.001). These findings indicate that EA treatment can improve cerebral ischemic injury and exert neuroprotective effects by attenuating neuronal damage via mTOR.

### 3.3. EA Intervention Increased Neuroplasticity-Related Proteins

We used Western blotting to detect neuroplasticity-related proteins, including GAP-43 and SYN (Figure 2(b)). As shown in Figure 2(d), GAP-43 protein expression was slightly increased in the p-MCAO group compared with the Sham group, but the difference was not statistically significant. GAP-43 protein expression was significantly increased in the EA group compared with the p-MCAO group (*p* < 0.05). As shown in Figure 2(e), SYN expression was significantly increased in the EA group compared with the p-MCAO group (*p* < 0.01); this positive effect was counteracted by rapamycin and the expression of SYN was significantly lower in the EA + R group compared with the EA group (*p* < 0.01). These results show that EA after p-MCAO increases the expression of GAP-43 and SYN in the contralateral cerebral cortex, suggesting that EA intervention after p-MCAO can help drive motor function recovery by enhancing neuroplasticity.

### 3.4. Relationship between Neuroplasticity-Related Proteins and the mTOR Pathway

**Figure 3:**
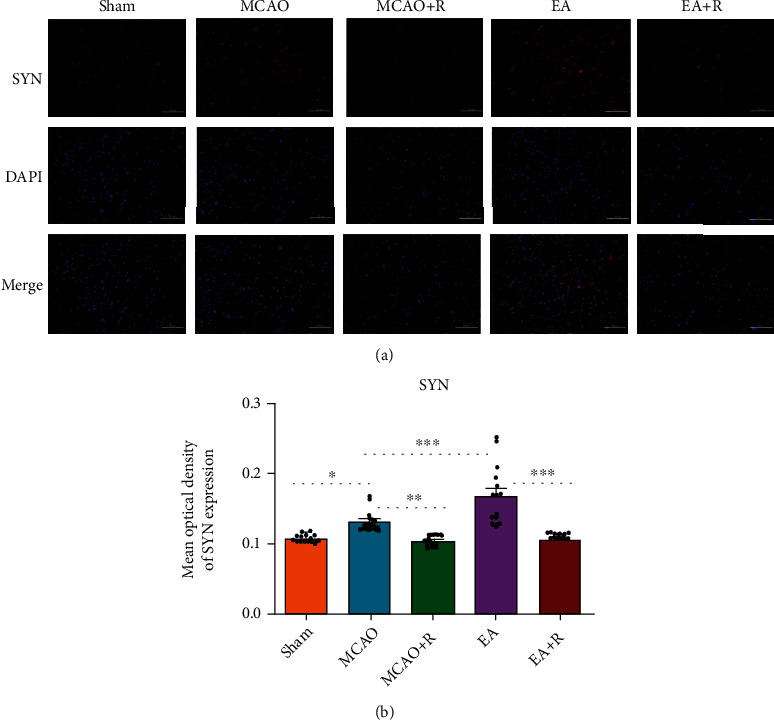
IF analysis of the effects of EA on SYN expression in the contralateral cerebral cortex. (a) IF analysis showed the expression of SYN^+^ in each group (*n* = 5). (b) The mean optical density of SYN in each group (^∗^*p* < 0.05, ^∗∗^*p* < 0.01, ^∗∗∗^*p* < 0.001, SYN (red) and DAPI (blue); scale bar = 100 *μ*m).

**Figure 4:**
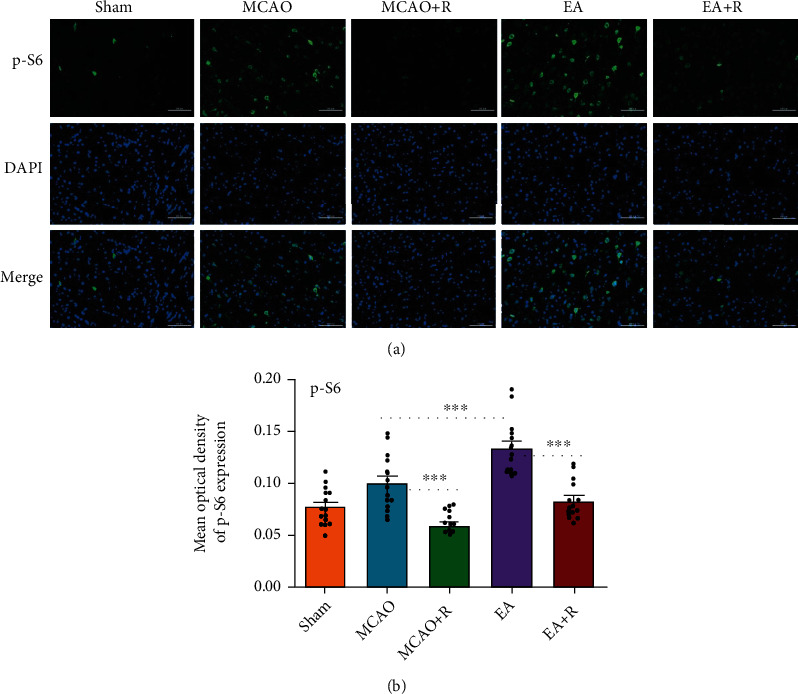
IF analysis of the effects of EA on p-S6 expression in the contralateral cerebral cortex. (a) IF analysis of p-S6^+^ in each group. (b) The mean optical density of p-S6 in each group (^∗^*p* < 0.05, ^∗∗^*p* < 0.01, ^∗∗∗^*p* < 0.001, p-S6 (green) and DAPI (blue); scale bar = 100 *μ*m).

**Figure 5:**
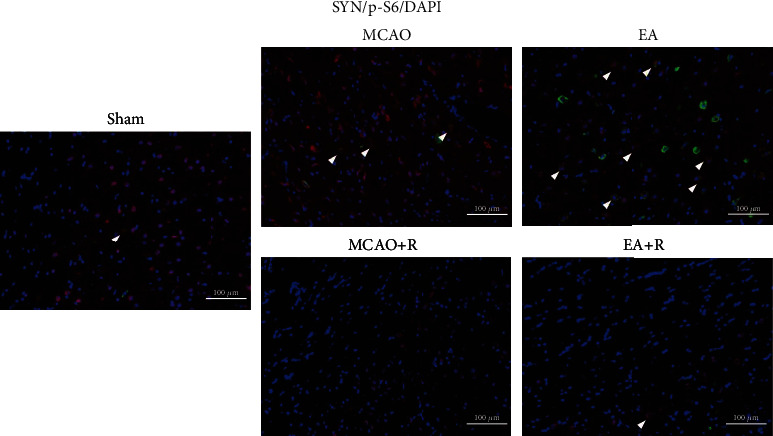
IF analysis revealed differential colocalization of p-S6^+^/SYN^+^ expression in the contralateral rat cerebral cortex. Arrows indicate positive cells, SYN (red), p-S6 (green), DAPI (blue), and co-SYN and p-S6 (yellow); scale bar = 100 *μ*m.

To further confirm the relationship between neuroplasticity-related proteins and the mTOR pathway, we detected the expression of SYN and p-S6 by immunofluorescence (Figures 3(a) and 4(a)). As shown in Figure 3(b), SYN expression was significantly increased in the p-MCAO group compared with the Sham group (*p* < 0.05), SYN was more increased in the EA group compared with the p-MCAO group (*p* < 0.001), lower in the p-MCAO + R than the p-MCAO group (*p* < 0.01), and lower in the E + R than the EA group (*p* < 0.001). As shown in Figure 4(b), p-S6 expression was significantly increased in the EA group compared with the p-MCAO group (*p* < 0.001) but decreased in the p-MCAO + R group (*p* < 0.001). In addition, p-S6 expression in the p-MCAO + R group was significantly lower than in the p-MCAO group (*p* < 0.001) and lower in EA + R than in the EA group (*p* < 0.001). Double immunofluorescence showed that p-S6 was present around SYN in the healthy cerebral cortex; these markers were even found to be coexpressed. Such SYN^+^/p-S6^+^ coexpressing cells were significantly increased after EA (Figure 5). This suggests that SYN correlates with p-S6 and that EA can modulate neuroplasticity through the mTOR pathway.

### 3.5. EA Activates mTOR and PTEN Pathways in the P-MCAO Healthy Cortex

**Figure 6:**
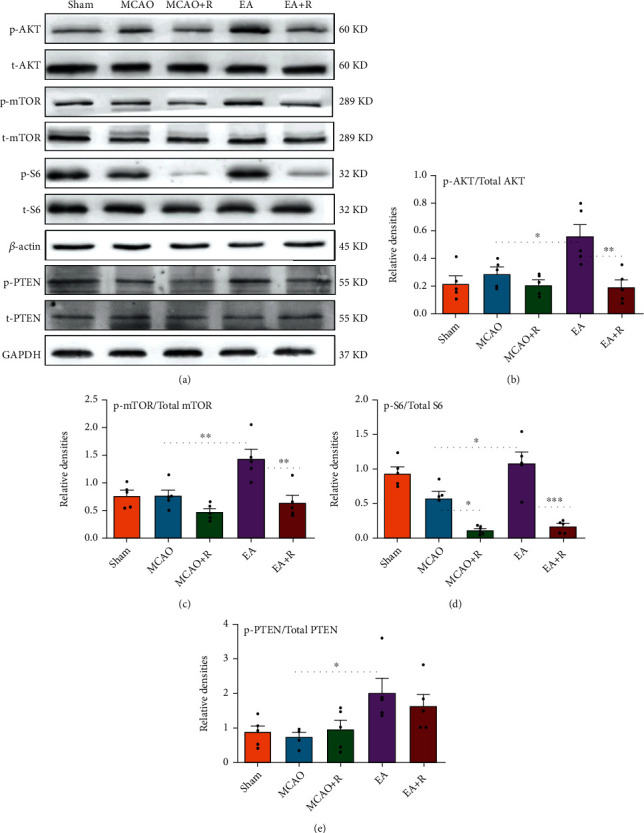
Effects of EA on mTOR and PTEN pathway proteins in the robust cortex of rats with cerebral ischemic injury. (a) Western blot analysis showing the levels of p-AKT, t-AKT, p-mTOR, t-mTOR, p-S6, t-S6, p-PTEN, and t-PTEN between the sham, p-MCAO, p-MCAO + R, EA, and EA + R groups (*n* = 5). (b) Statistical analysis showing the levels of p-AKT/t-AKT in the cortex cerebral cortex. (c) Statistical analysis showing the levels of p-mTOR/t-mTOR in the cerebral cortex. (d) Statistical analysis showed the levels of p-S6/t-S6 in the cerebral cortex. (e) Statistical analysis showing the levels of p-PTEN/t-PTEN in the cortex cerebral cortex (^∗^*p* < 0.05, ^∗∗^*p* < 0.01, ^∗∗∗^*p* < 0.001).

To test how EA affects the mTOR and PTEN pathways after ischemic stroke, we used Western blotting to detect total AKT (t-AKT), phosphorylated AKT (p-AKT), total mTOR (t-mTOR), phosphorylated mTOR (p-mTOR), total S6 (t-S6), phosphorylated S6 (p-S6), total PTEN (t-PTEN), and phosphorylated PTEN (p-PTEN) expression (Figure 6(a)). As shown in Figure 6(b), AKT phosphorylation (p-AKT/t-PAK) levels were significantly higher in the EA compared to the p-MCAO group (*p* < 0.05), while AKT phosphorylation was significantly lower in the EA + R compared to the EA group (*p* < 0.01). As shown in Figure 6(c), the mTOR (p-mTOR/t-mTOR) phosphorylation levels in EA were significantly higher than in the p-MCAO (*p* < 0.01) and EA + R groups (*p* < 0.01) after EA. Although the mTOR phosphorylation level in the p-MCAO + R group was slightly lower than that in the p-MCAO group, the difference between the groups was not statistically significant (*p* > 0.05). As shown in Figure 6(d), the level of S6 phosphorylation (p-S6/t-S6) was slightly decreased in the p-MCAO compared with the Sham group but the difference was not significant. After EA intervention, the level of S6 phosphorylation was significantly higher in the EA than in the p-MCAO group (*p* < 0.05), while the EA + R group counteracted this change (*p* < 0.001), and the p-MCAO + R group had a significantly lower S6 phosphorylation level than the p-MCAO group (*p* < 0.05). These results suggest that EA can exert neuroprotective effects on p-MCAO rats by modulating the mTOR pathway.

PTEN is a negative regulator of the mTOR pathway. Using Western blot analysis (Figure 6(e)) we found that the level of PTEN phosphorylation (p-PTEN/t-PTEN) was significantly higher in the EA than in the p-MCAO group after EA. The activity of PTEN is known to be inhibited after phosphorylation [21]. This indicates that EA can alter the negative regulatory effect of PTEN on the mTOR pathway and produce further mTOR pathway activation by inhibiting the PTEN protein.

### 3.6. EA Promotes Axonal Sprouting in the CST

**Figure 7:**
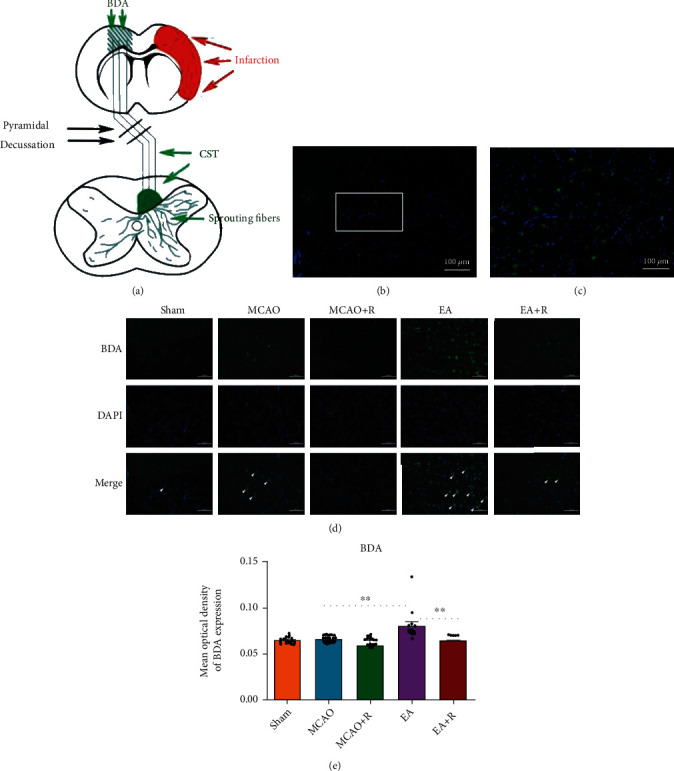
Effect of EA intervention on CST axon regeneration. (a) Schematic diagram of BDA cis-neural tracer staining. (b) BDA labeling in the anterior horn region of the spinal cord gray matter in the EA group. (c) This image is a partial zoom of the white box in (b). (d) Expression of BDA-positive cells in the cervical medullary gray matter in each group (*n* = 5). (e) Statistical analysis of BDA-positive cells in the cervical medullary gray matter of each group (^∗^*p* < 0.05, ^∗∗^*p* < 0.01, ^∗∗∗^*p* < 0.001, arrows indicate positive cells, BDA (green), DAPI (blue); scale bar = 100 *μ*m).

**Figure 8:**
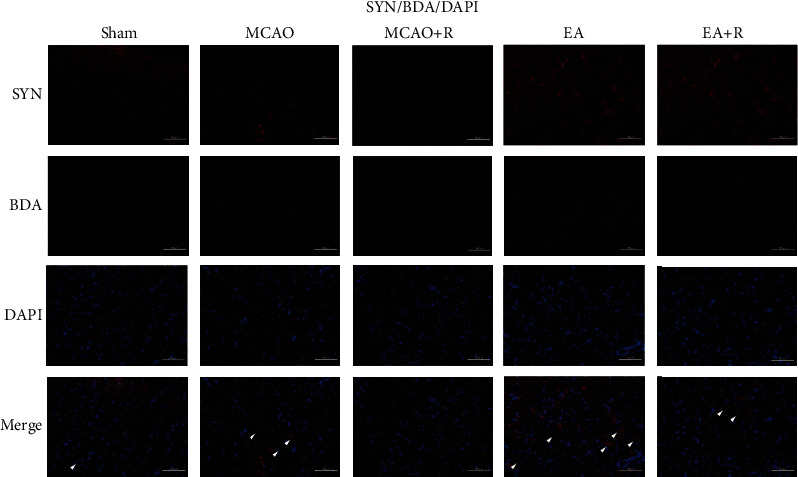
The relationship between SYN and axonal sprouting in the gray matter region of the cervical medulla. Expression of BDA^+^/SYN^+^ in the cervical medullary gray matter in each group (^∗^*p* < 0.05, ^∗∗^*p* < 0.01, ^∗∗∗^*p* < 0.001, arrows indicate positive cells, BDA (green), SYN (red), DAPI (blue), and co-BDA and SYN (yellow); scale bar = 100 *μ*m).

**Figure 9:**
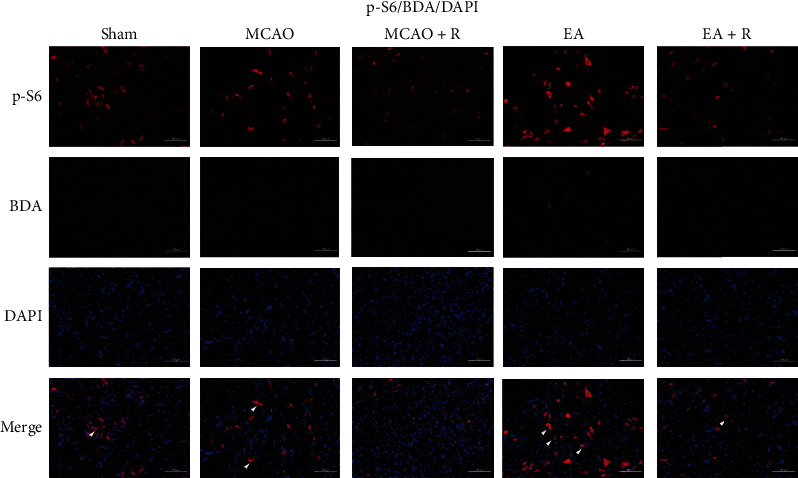
Expression of BDA^+^/p-S6^+^ in the cervical medullary gray matter in each group (^∗^*p* < 0.05, ^∗∗^*p* < 0.01, ^∗∗∗^*p* < 0.001, arrows indicate positive cells, BDA (green), p-S6 (red), DAPI (blue), and co-BDA and p-S6 (yellow); scale bar = 100 *μ*m).

To further investigate the relationship between axonal sprouting and neurological recovery in the contralateral CST, we injected BDA into the cortex contralateral to the infarct to track the CST and observed the changes and development following EA (Figure 7(a)). We observed activation of endogenous remodeling after ischemic injury and found that a large number of axonal sprouts appeared in the CST in the C1–C4 spinal cord region gray matter after EA, further enhancing endogenous remodeling (Figures 7(b) and 7(c)). We then examined BDA labeling in each group by immunofluorescence (Figure 7(d)); as shown in Figure 7(e), BDA positive expression was significantly increased in the EA compared to the p-MCAO group (*p* < 0.01), while rapamycin reversed the EA group changes. BDA positive expression was significantly lower in the E + R than in the EA group (*p* < 0.01). In addition, we found that SYN and p-S6 were expressed around BDA markers; EA significantly increased the expression of BDA^+^/SYN^+^ and BDA^+^/p-S6^+^ positive cells (Figures 8 and 9), demonstrating that EA promotes SYN expression and activates CST axon germination in the cervical medullary gray matter via the mTOR pathway.

## 4. Discussion

Previous studies have shown that EA therapy can significantly improve neurological function after stroke [22]. In this paper, we first demonstrated the effects of EA on neurological deficits in p-MCAO rats and found that electroacupuncture significantly reduce mNSS neurological deficit scores, cerebral infarct volume, and neuronal damage in the motor cortex, thereby alleviating ischemic brain injury. The body activates neuroprotective mechanisms after ischemic stroke, including the promotion of cell proliferation and neurogenesis, regulation of cerebral blood flow and neurochemicals, and the inhibition of cell death in the ischemic area [23]. EA appears to enhance this and protects cerebral tissues from ischemic injury.

Several studies have shown that recovery of neurological function depends on CST neuroplasticity; specifically, the generation of new lateral branches and synaptic connections with newborn neurons in the vicinity of the cerebral ischemic region [24]; meanwhile, axonal growth from the healthy to the affected side is found in the gray matter of the contralateral cortex and spinal cord and these new connections promote some degree of motor recovery [25]. GAP-43, a neurotrophin-dependent membrane-bound phosphorylated protein, is expressed in axons in CNS regions that are undergoing plasticity [26]. It has also been demonstrated that the distribution and density of SYN can indirectly reflect the number and transmission efficiency of synapses and activate neuroplasticity [27]. Expression of GAP-43 and SYN in the postinfarction healthy cortex correlates with synapses, synaptic transmission, and neuroplastic activation [24]. EA pretreatment can increase the expression of GAP-43 and BDNF and alleviate ischemic injury and promote axonal regeneration, thereby providing protection for functional recovery following stroke [28]. It has been reported that PSD-95 and SYN expression is reduced in the CA1 region of the hippocampus after ischemic stroke and that this is reversed by EA. A large number of tightly arranged and clearly visible synapses were reported, supporting the positive effect of EA on neuroplasticity [29]. Similarly, we showed that 14 days after p-MCAO, EA significantly increased the expression of GAP-43 and SYN within the contralateral cortex, but only SYN expression was reversed by the mTOR inhibitor rapamycin. This demonstrates that EA exerts neuroprotective effects by activating the neuroplasticity-associated proteins GAP-43 and SYN. In addition, we found that SYN, but not GAP-43, plays a key role in the regulation of the mTOR pathway by EA, its mechanisms need to be further explored.

Several studies have shown that mTOR plays a key role in neuroprotection activated by EA in cerebral ischemia and the AKT-mTOR-S6 signaling axis has an important functional role in the intrinsic mechanisms of axonal regeneration [30, 31]. The signal upstream from mTOR, followed by phosphorylation of the downstream ribosomal protein S6 at serine 235/236, promotes protein translation and finally plays an important role in cell growth, proliferation, neuroplasticity, and axonal regeneration [32]. Simultaneously, the p-S6 protein expression increases significantly after stroke when neuronal repair begins. Therefore, the p-S6 protein is often used as a molecular marker to determine neuronal cell growth and cell activity status [33]. Studies have also shown a strong correlation between S6 phosphorylation levels and axonal growth [34]. In the present study, EA significantly increased the expression of p-AKT, p-mTOR, and p-S6; this was reversed by rapamycin. In addition, further detection by p-S6^+^/SYN^+^ double-labeled immunofluorescence assays showed that EA significantly increased the colocalized expression of p-S6 and SYN in the cerebral cortex after ischemic stroke, suggesting that EA activation of the neuroplasticity-related protein SYN may be related to the mTOR signaling pathway.

PTEN is a negative regulator of mTOR [35]. It has been shown that Pten knockdown increases the ability of the mTOR-dependent signaling pathway to activate axonal regeneration [33, 36]. Western blot analysis showed that EA significantly increased the phosphorylation level of PTEN after ischemic stroke. Studies have shown that when PTEN factors are phosphorylated, activity can be inhibited [21]. This suggests that EA may produce further activation of the mTOR signaling pathway by inhibiting PTEN activity.

Following maturation, neurological function is largely dependent on the CST, which contains the direct projections from the motor cortex of the brain to the spinal cord, as well as the brainstem motor pathway [4]. After a brain or spinal cord injury, the axons of the CST on the healthy side can pass through the growing lateral branches to the affected side to aid in neurological functional recovery [37]. To determine the CST and its projections, Huang injected BDA tracers into the contralateral motor cortex after MCAO. Positive BDA signals were detected in the cervical medullary gray matter (C3-5) with extended germination across the midline into the infarcted side [38]. Deng further confirmed that EA increases the number of positive BDA signals on the infarct side [10]. In our study, electroacupuncture similarly increased the number of positive BDA signals in the cervical medullary gray matter and was reversed by rapamycin. In addition, we found that a large amount of SYN and p-S6 fluorescent signals were present around BDA-positive markers in the gray matter region of the spinal cord in the EA group, with some coexpression being evident. These results suggest that EA treatment enhanced CST axon regeneration after stroke; this may be related to EA stimulation and amplification of endogenous recovery mechanisms after brain injury and promotion of brain remodeling.

**Figure 10:**
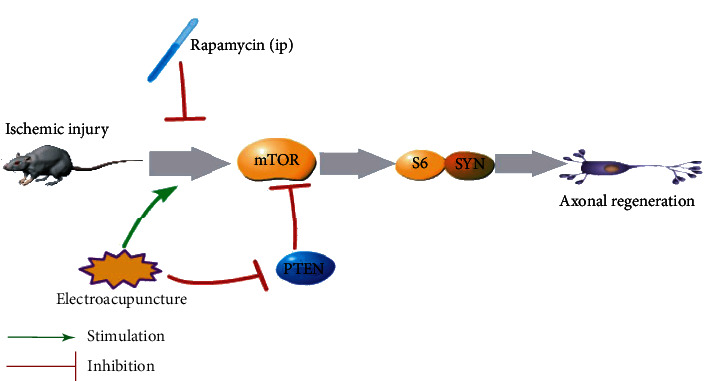
Diagram depicting the signaling mechanisms of electroacupuncture in ischemic brain injury. In rats with ischemic brain injury, electroacupuncture activates the mTOR pathway directly and by inhibiting PTEN; this effect is reversed by the mTOR inhibitor rapamycin. These electroacupuncture effects activate the expression of downstream S6 and SYN proteins to promote axon regeneration.

Axonal regeneration, as an important mechanism for restoring neurological function after ischemic brain injury, is becoming a rapidly developing field [39]. In other studies, catalpol has been shown to activate mTOR and its downstream S6 protein, thereby inducing cellular activity and ultimately activating axonal regeneration after stroke [31]. In addition, EA has been shown to enhance neuroplasticity in hippocampal CA1 area pyramidal cells after ischemic stroke, promoting neural recovery [40]. EA also attenuates cerebral ischemic injury by inhibiting neuronal apoptosis and autophagy through the activation of the mTOR pathway [15]. According to these results, we demonstrated that mTOR is essential for CST axon sprouting and that EA may promote CST axon regeneration through the mTOR pathway to achieve a neuroprotective effect (Figure 10).

## 5. Conclusion

In summary, we determined that activation of the mTOR signaling pathway and increased expression of GAP-43 and SYN after stroke are beneficial to axonal regeneration and neurological recovery. The mechanism of electroacupuncture treatment may be related to promoting axonal regeneration in the motor cortex and CST contralateral to the infarct by regulating the mTOR pathway, activating neuroplasticity, and ultimately improving neurological dysfunction.

## Data Availability

The datasets used and/or analyzed during the current study are available on reasonable request from the corresponding author.
